# Supplementary material for: That’s My Cue to Eat: A Systematic Review of the Persuasiveness of Front-of-Pack Cues on Food Packages for Children vs. Adults
Source: Nutrients. 2020 Apr 11;12(4):1062. doi: 10.3390/nu12041062 (PMC7230775; doi:10.3390/nu12041062)
Supplement: Supplementary file 1 [file nutrients-12-01062-s001.zip › S1. Search string.pdf]

Domain	Search words	
**Population**	(Consumer*	OR
	customer*	OR
AND	child*	OR
	adolescent*	OR
	adult*	OR
	senior*	OR
	people*)	
**Intervention: Package**	(food	OR
	beverage	OR
AND	drink	OR
	snack*)	
	AND	
	(front-of-pack*	OR
	FOP	OR
	On-pack	OR
	packag*)	
**Intervention: Cues**	(marketing	OR
	visual	OR
	sensory	OR
AND	textual	OR
	design)	
	AND	
	(cue*	OR
	attribute*	OR
	technique*	OR
	element*	OR
	label)	
	(color*	OR
	colour*)	
	typograph*	OR
	(endorse*	OR
	celebrit* endorse*)	

	OR<br><br>((cartoon* OR<br>licensed OR<br>fictional OR<br>brand)<br><br>AND<br><br>character* OR<br>figure)
	(convenience OR<br>health OR<br>nutrition* OR<br>carbon OR<br>sutainab* OR<br>environment*)<br><br>AND<br><br>(information OR<br>message* OR<br>claim* OR<br>warning)
	(health OR<br>calori* OR<br>nutrition OR<br>eco* OR<br>Sutainab* OR<br>environmental*<br>serving size<br>food)<br><br>AND<br><br>(label* OR<br>symbol* OR<br>table)<br><br>OR<br><br>OR<br>(traffic light*

	nutri-score)	
	(product<br>portion<br>food<br>ingredient<br>nutrient)<br><br>AND<br><br>(picture*<br>illustration*<br>size*<br>imag*<br>presentation*)	OR<br>OR<br>OR<br>OR<br><br><br><br><br>OR<br>OR<br>OR<br>OR
	(Brand<br>product)<br><br>AND<br><br>(logo*<br>slogan*<br>baseline*<br>tagline*<br>name*)	OR<br><br><br><br><br>OR<br>OR<br>OR<br>OR
	(size*<br>partition*<br>serving*)	OR<br>OR
	shape*	
	(material*<br>transparent<br>squeeze)	OR<br>OR
	seal*	OR
	call-to-action*	OR
	game*	OR
	competition*	OR

	Collection*	OR
	((price<br>pricing<br>premium<br>quantity)<br><br>AND<br><br>(promotion*<br>discount*)<br><br>OR<br><br>(gift*<br>toy*<br>Premium))	OR<br>OR<br>OR<br><br><br><br>OR<br><br><br>OR<br>OR
**Outcome**<br><br>AND	((food*<br>beverage*<br>drink<br>snack*)<br><br>AND<br><br>(selection*<br>choice*<br>intake*<br>consumption*<br>amount*<br>behavior?*<br>purchase*))<br><br>OR<br><br>energy*<br>diet<br>calori*<br>eat*<br>consum*	OR<br>OR<br>OR<br><br><br><br><br>OR<br>OR<br>OR<br>OR<br>OR<br>OR<br><br><br><br><br>OR<br>OR<br>OR<br>OR
**Study design**	(experiment*<br>RCT	OR<br>OR

	randomi?ed controlled trial	OR
	conjoint analys!s	OR
	intervention*	OR
	lab study)	
**Excluded terms**	(bacteri*	OR
	HIV	OR
NOT	pharmaceutical	OR
(in title / abstract)	antimicrob*)	
