# Supplementary material for: That’s My Cue to Eat: A Systematic Review of the Persuasiveness of Front-of-Pack Cues on Food Packages for Children vs. Adults
Source: Nutrients. 2020 Apr 11;12(4):1062. doi: 10.3390/nu12041062 (PMC7230775; doi:10.3390/nu12041062)
Supplement: Supplementary file 1 [file nutrients-12-01062-s001.zip › S2. Additional results.pdf]

Supplementary results

1. Visual cue

1.1 Branding

Four studies assessed the impact of branding on the food behaviors of children (age range: 4-9 years old; n = 2) and adults (n = 2). Only one study concluded that branding directly influenced food behavior, more specifically that adults were more likely to choose a product from a well-known brand compared to a lesser known private-label brand [43]. In three other studies, children (n = 2) and adults (n = 1) consumed similar amounts of core and non-core food products when the foods were linked to a well-known brand as when they were presented in plain packaging or no packaging. However, two studies did uncover an interaction effect. Forman et al. [59] found that overweight children consumed more calories from branded food packages compared to normal-weight children. Similarly, Keller et al. [45] found that girls but not boys consumed more calories from branded packages.

1.2 Package shape

In three studies, Huyghe et al. [57] measured the impact of package shape and found an effect on the amount of food people serve themselves. Non-core food products such as butter and mayonnaise were provided in traditional or in squeezable packages. Participants were instructed that they could consume the products later on, but measures of actual intake were not included. In all studies, people served themselves more non-core products when the food was presented in a squeezable package.

1.3 Product visibility

Two studies investigated the impact of product visibility and found an effect on product choice or intake. Gomes et al. [60] found that adults are more likely to choose a beverage when the package is partially covered, allowing consumers to see the liquid, compared to when the package is completely covered. Argo and White [41] also found that adults consume more when the product is visible through transparent packaging compared to when the product is covered.

1.4 Sealability

In two studies, De Bondt et al. [33] found that people eat less non-core foods when these foods are presented in a sealable package. This effect occurred in the short term (i.e. during one film viewing) as well as in the longer term (i.e. during a six-day period at home).

2. Informational cue

2.1 Claim

Three studies investigated whether one or more written claims impact adults' product choices and consumption amounts. Two studies (i.e. 50.0%) concluded that a claim directly influenced behaviors. Koenigstorfer et al. [75] found that adults consumed more core foods from a package that contained a 'fitness' claim. Aschemann-Witzel and Hamm [77] also found that people are more likely to choose a product with a claim compared to packages without a claim. Finally, in the study by Steinhauser et al. [76], products displayed either a nutrition claim, a health claim or a taste claim. The claims had an effect on food behaviors, depending on the type of food. For core products, a nutrition claim positively influenced product choices, whereas health and taste claims had no effect. For non-core food products, only a taste claim positively influenced product choice.

2.2 Congruency cue

Gutjar et al. [78] found that package information signaling that a product was consistent or inconsistent with the consumption context influenced adults' product choices. This cue influenced adults' product choices. Specifically, packages contained claims and information signaling that the

product was (in)appropriate to consume for breakfast. Adults either tasted the product, saw the package or both. When asked to choose a product for breakfast, participants chose the appropriate product more often when they could see the packaging compared to when they only tasted the product.
